# Multidrug-resistant strains of *Mycobacterium* complex species in Egyptian farm animals, veterinarians, and farm and abattoir workers

**DOI:** 10.14202/vetworld.2020.2150-2155

**Published:** 2020-10-14

**Authors:** Hossam A. Abdelsadek, Hassan M. Sobhy, Kh. F. Mohamed, Sahar H. A. Hekal, Amany N. Dapgh, Ashraf S. Hakim

**Affiliations:** 1 Central Administration of Veterinary Quarantine, General Organization for Veterinary Services, Dokki, Giza, Egypt; 2 Department of Natural Resources, Faculty of African Postgraduate Studies, Cairo University, Cairo, Egypt; 3 Department of Microbiology, Faculty of Veterinary Medicine, Cairo University, Cairo, Egypt; 4 Department of Bacteriology, Animal Health Research Institute, Dokki, Giza, Egypt; 5 Department of Microbiology and Immunology, National Research Centre, Dokki, Cairo, Egypt

**Keywords:** antituberculous drugs, Delta Egypt, multidrug-resistant tuberculosis, *Mycobacterium tuberculosis* complex

## Abstract

**Background and Aim:**

*Mycobacterium tuberculosis* complex (*MTBC*) is a group of mycobacteria that are important human pathogens. *Mycobacterium tuberculosis* and *Mycobacterium bovis* cause serious chronic life-threatening disease and also significant economic losses in both production and remedication. Recently, emergence of multidrug-resistant tuberculosis (MDR-TB) complex has generated global recognition of the need for rapid and sensitive diagnosis and development of new treatments. The current study illustrates the isolation/identification of *MTBC* strains in specimens obtained from cows and humans by conventional and real-time polymerase chain reaction (RT-PCR) techniques. Further, the study assesses sensitivity to antituberculosis drugs in isolated MDR strains.

**Materials and Methods:**

A total of 1464 samples from cattle (1285 raw milk and 179 lymph node), and 149 human sputum samples, were collected from farms and abattoirs in Delta Egypt. Conventional methods (culture and Ziehl–Neelsen staining) were implemented as were RT-PCR using *MTBC* universal DNA. The effect of some antituberculosis drugs on obtained isolates was assayed using drug susceptibility proportion and qualitative suspension techniques.

**Results:**

The MBTC detection rate using the culture method was higher than for Ziehl–Neelsen staining; raw cow milk (2.56 vs. 1.63%), lymph nodes (51.59 vs. 48.04%), and human sputum (5.36 vs. 4.02%). A total of 135 isolates were obtained. Application of RT-PCR detected 138 isolates from the same set of samples. MBTC isolates were resistant to first-line antituberculosis drugs, such as pyrazinamide, isoniazid, rifampicin, and ethambutol by 78.5, 59.3, 40.7, and 31.8%, respectively, and could be highly resistant to kanamycin (82.3%) and amikacin (80.7%). However, isolates remained sensitive to ciprofloxacin (71.1%) and clarithromycin (73.3%) as second-line drugs.

**Conclusion:**

There is a growing risk for isolation of MDR-TB from raw milk and lymph nodes of field tuberculin positive cattle as well as sputum of veterinarians and workers existed in farms and abattoirs. PCR-based techniques have become the gold standard for the identification of mycobacterial species, showing high efficiency compared to bacteriological and microscopic examination. Application of the first- and second-line antituberculosis drugs in combination could counter the MDR-TB concern once infections are identified.

## Introduction

Human tuberculosis (TB) is a contagious and infectious disease primarily caused by *Mycobacterium tuberculosis*, a member of what is termed *Mycobacterium tuberculosis* complex (*MTBC*). The organism is an aerobic pathogenic bacterium that typically causes respiratory infection. TB displays epidemic cycles that might, rarely, last for centuries [1]. Conversely, bovine tuberculosis is a chronic granulomatous disease of cattle caused by *Mycobacterium bovis*, another member of the complex. This disease has a socioeconomic significance due to impacts on international trade in animals and animal products, the potential for human infection [2]. Human infection may be caused by drinking unpasteurized milk, eating undercooked meat, and close contact with infected animals. Patients may suffer chest pain, coughing up blood, recurrent and constant cough, night sweats, fever, fatigue and weakness, loss of appetite, and pain with breathing or coughing. Further, lymph nodes near the heart and lungs become enlarged [3]. The emergence of AIDS-associated infections and other immunodeficiency diseases has coincided with increasing mycobacterial infections. Compromised immunity increases susceptibility to infection. Otherwise, the critical concern is the appearance of multidrug-resistant tuberculosis (MDR-TB) strains that encourage increasing incidence and severity of the disease [4].

TB is usually treated with antimicrobial agents with a course of drug therapy that usually lasts from 6 to 9 months. Common first-line drugs are rifampin, isoniazid (INH), pyrazinamide (PZA), ethambutol (EMB), and streptomycin (SM). The second-line drugs include other aminoglycosides and the new class of fluoroquinolones. Antimycobacterial drugs are not used singly; TB has been treated with combination therapy for over 50 years. Treatment regimens that use only single drugs result in the rapid development of resistance and treatment failure [5]. MDR-TB strains are resistant to at least two of first-line anti-TB drugs. Extensively drug-resistant TB (XDR TB) is relatively rare and involves resistance to INH and rifampicin, plus resistance to any fluoroquinolone and at least one of three injectable second-line drugs, amikacin, kanamycin, or capreomycin. XDR TB is resistant to both the first-line and second-line drugs, and patients have fewer effective treatment options; moreover, **t**reatment is often less effective [6]. Development of new drug combinations to augment the efficacy of common drugs is needed to combat resistant strains [7].

This study aimed to examine the incidence of *M. tuberculosis* complex in human and animal samples and assess resistance in isolated strains to some antimycobacterial agents.

## Materials and Methods

### Ethical approval

As per CPCSEA guidelines, a study involving clinical and postmortem samples does not require the approval of the Institute Animal Ethics Committee.

### Study period and location

Samples were collected from the Egyptian Delta governorates of El-Sharkia, El-Menofia, and El-Gharbiya from September 2018 to April 2019 (Table-1).

**Table 1:** The distribution of collected samples (its number and type) in concern to its source.

Sample/origin	Human	Cattle	Total
Sputum samples	149	-	149
Milk samples	-	1285	1285
Lymph nodes	-	179	179
Total			1613

### Sampling

A total of 1613 samples were collected from human (149) and bovine (1464) sources. Raw milk samples were collected in sterile containers after cleaning and washing udders from adult (5-9 years) tubercular dairy cows. Total numbers of animals in this region are about 25,000 with an average of 1200 animals per farm. Infected animals suffered from emaciation, lethargy, weakness, anorexia, low-grade fever, and pneumonia with a chronic, moist cough, accompanied by enlarged lymph nodes. The hepatic, intestinal, renal, and supramammary lymph nodes were collected under completely hygienic conditions from tuberculin positive cattle after the slaughter in El-Sharkia (4 of 22 abattoirs), El-Menofia (8 of 32 abattoirs), and El-Gharbiya (4 of 18 abattoirs).

The sputum samples were obtained from all employees (veterinarians and workers) at the above abattoirs. Three morning sputum specimens were collected on 3 consecutive days from each individual. In Egypt, the BCG vaccination is obligatory at the age of 1 year. All samples were kept in clean, tightly closed labeled plastic disposable containers and transported in iceboxes to the laboratory as soon as possible.

### Isolation and identification of acid-fast bacilli

After preparation, the samples were cultivated in modified Lowenstein–Jensen media in labeled McCartney tubes with incubation at 37°C. Cultures were examined daily for 7 days, then periodically once a week for up to 6-8 weeks. The type and rate of growth were recorded [8]. Direct smears were made from isolated colonies, fixed by gentle heating, stained using the Ziehl–Neelsen method, and examined microscopically and biochemically for acid-fast organisms.

### Molecular diagnosis of *M. tuberculosis* complex

Samples from tuberculin positive cows were examined by real-time polymerase chain reaction (RT-PCR). DNA was extracted following extraction kit instructions (Sigma-Aldrich, USA). Real-time PCR was performed using MTplexdtec-RT-qPCR Test (Edificio-Quórum3, Spain) that comprises a series of species-specific targeted reagents designed for the detection of all species of the *MTBC* (Table-2) [9]. Reaction was run using an Applied Biosystem StepOne RT-PCR System. FAM fluorogenic reporter dyes were used to establish a threshold for reactions using StepOne™ software version 2.2.2 (Life Technologies, Thermo Fischer, USA). The threshold cycle was defined as 10 times the standard deviation of mean baseline fluorescence emission calculated for PCR cycles 3-15. For a sample to be considered positive, the corresponding amplification curve had to exhibit three distinct phases (geometric, linear, and plateau) that characterize the progression of the PCR reaction.

**Table 2:** Real-time PCR: Primer sequences, target gene, amplicon sizes, and cycling conditions.

Sequence	Target gene	Amplified segment (bp)	Hybridization	Extension	Data collection	Reference
INSI (5’CGTGAGGGCATCGAGGTGGC 3’) INS2 (5’GCGTAGGCGTCGGTGACACAAA 3’)	*16S rDNA*	143	95°C for 5 min one cycle	95°C for 0.5 min 45 cycles	60°C for 1 min one cycle	[9]

### Antimycobacterial drug susceptibility proportion assay

The susceptibility of the isolates to various antibiotics was assessed using the drug proportion diffusion technique. Disks containing INH (0.2 μg), PZA (30 μg), EMB (2 μg), and rifampicin (40 μg) were used to test first-line drugs. The second-line drugs, kanamycin (40 μg), amikacin (10 μg), ciprofloxacin (30 μg), and clarithromycin (30 μg), were also tested [10].

## Results and Discussion

Mycobacteria are found commonly in nature and remain an important cause of infections in human and animals worldwide. The *MTBC* is a group of closely related pathogens that induce TB in mammalian species. The constituent members of the *MTBC* are divided into human and animal-adapted strains, *M. tuberculosis* and *M. bovis* [11].

*M. tuberculosis* is the predominant cause of human TB cases, a significant portion 0.5-7.2% in developed countries and 10-15% in developing countries–is due to *M. bovis* infection. This disease is considered zoonotic, and person-to-person transmissions are not reported [12]. Zoonotic bovine TB is the most common reason for recurrent TB in humans. The disease is typically acquired through consumption of unboiled or unpasteurized milk and dairy products. Unpasteurized raw milk is preferably consumed in developing countries, especially in Africa and the Middle East, due to its accessibility, suitability, palatability, and lower cost [13].

Rapid detection, isolation, identification, and susceptibility testing of mycobacteria isolates from clinical specimens are necessary for control/prevention of disease [14]. As shown in Table-3, it was found that MBTC was successfully isolated from raw milk (33/1285), lymph nodes (94/179), and employee sputum (8/149), with an incidence of 2.56%, 51.59%, and 5.36%, respectively. Microscopic examination of positive acid-fast bacilli showed fewer detections – raw milk (21/1285), lymph nodes (86/179), and employee sputum (6/149), with an incidence of 1.63%, 48.04%, and 4.02%, respectively. Culture of isolates from lymph nodes of tuberculin positive animals was superior to direct microscopic examination. This observation is consistent with other studies that attributed results to the numbers of bacilli in examined samples [15,16].

**Table 3:** The prevalence rate of mycobacteria by conventional methods.

Source	No. of samples	Bacteriological findings			

		Cultivation finding		Microscopical findings	
	
		Number of isolates	%	Number of isolates	%
Cattle raw milk	1285	33	2.56	21	1.63
Cattle lymph nodes	179	94	51.59	86	48.04
Human sputum	149	8	5.36	6	4.02

Furthermore, data from raw milk samples collected from infected dairy cows in the farms revealed that the positive Ziehl–Neelsen specimens were lower than the culture-positive isolates. This isolation percentage is close to that recorded in Tunisia (4.9%) [9], but greatly lower than that reported in Morocco (18-33%) [13]. This low incidence may be attributed to the small quantity of produced milk that is sold at retail and may be consumed raw or used for fermented dairy products. Conversely, the exaggerated percentage of isolation in Morocco is likely due to raw milk samples representing bulk tank milk contaminated by relatively few infected cows [17].

Culture from sputum samples collected from the workers (veterinarians and workers) on Lowenstein–Jensen media revealed eight positive isolates (5.5%) of *Mycobacterium* spp.; microscopic examination of sputum samples using Ziehl–Neelsen stain identified only six positive samples (4.02%). These findings are consistent with similar investigations [18,19]. Our data indicate that culture methods are more sensitive than the microscopic examination to detect mycobacteria in clinical specimens. Culture methods may detect as few as 10^1^-10^2^ organisms/ml in a single specimen [20].

The risk of disease spread, the emergence of drug-resistant strains, and the severity of disease in immunocompromised patients necessitate prompt diagnosis of *M. tuberculosis* complex. Rapid detection of active TB infection is critical for the identification of new cases, efficient patient management, implementation of infection control measures, and development of appropriate antimycobacterial therapy [21].

Genomic sequence analysis uses PCR as the basis for most work in molecular biology. *In* *vitro* amplification of specific sequences of pathogen genomes allows rapid diagnosis with greater sensitivity and specificity than slower and laborious conventional methods. RT-PCR methods are based on hybridization of amplified nucleic acids with fluorescent-labeled probes spanning DNA regions of interest and monitoring of reactions with thermal cyclers. The main advantages of RT-PCR methods are its speed in producing results, 1.5-2.0 h after DNA extraction, and lower risks of contamination since both reaction and detection occur in a single tube [22].

**Figure-1:**
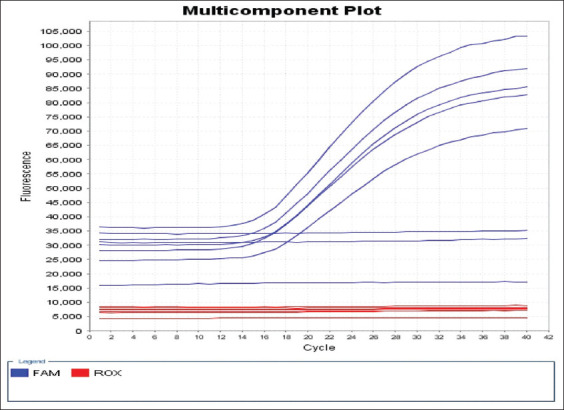
The amplification blot of tuberculous samples. Analysis for the amplification blot in its linear form: This photo consisted of four positive samples at cycle 12 and one control positive sample. There are three negative samples. The used reference dye is FAM. The run is for 45 cycles.

Our study used 138 positive samples for the detection of *MTBC* from 179 lymph nodes samples with RT-PCR to target universal bacterial *16S rDNA*. Positive amplification of target primers was seen in 77.09% of samples in real-time PCR (Figure-1). These data show more positive detections than conventional culture (51.59%) or microscopy (48.04%). This result is consistent with the previous reports [23,24] and supports the concept that PCR is a sensitive screening assay for the detection of *MTBC* [25].

The first-line antituberculosis drugs are mainly bactericidal and display a high efficacy with relatively low toxicity to patients during treatment. These agents include PZA, rifampicin, SM, INH, and EMB. Conversely, the second-line antituberculosis drugs are mainly bacteriostatic, less efficacious, and usually more toxic, for example, ciprofloxacin, kanamycin, amikacin, and clarithromycin [26]. Effective TB chemotherapy must include early bactericidal action against rapidly growing organisms and subsequent sterilization of dormant populations of bacilli. The first-line antituberculosis drugs exhibit early bactericidal activity against actively metabolizing bacilli and bacteriostatic second-line antituberculosis drugs are reserved to improve efficacy in the presence of resistance [27]. Occasionally, initial resistance to INH is encountered, necessitating the addition of another first-line drug, typically rifampicin. If resistance occurs to both drugs later in therapy, the bacterium has become an MDR-TB strain. Successful treatment requires extended time and frequent reliance on the use of second-line drugs [28]. Very recently, there is a new class of MDR, which was named extensively drug-resistant (XDR) TB; whose isolates are resistant to isoniazid and rifampicin and at least three of the six main classes of second-line drugs (thioamides, cycloserine aminoglycosides, polypetides, fluoroquinolones, and para-aminosalicylic acid) [29].

As shown in Table-4, the resistance of MBTC isolates was high for both PZA and INH, 78.5% and 59.3%, respectively. This incidence of high resistance may be attributed to the availability of drugs in the private market and widespread use as antibacterial agents [30,31]. In contrast, resistance to EMB and rifampicin was 31.8% and 40.7%. EMB maintains important efficacy as an antituberculosis drug and is often used to treat drug-resistant TB [32,33]. MBTC isolates were highly resistant to the second-line drugs, kanamycin 82.3% and amikacin 80.7%, but were sensitive to ciprofloxacin and clarithromycin, 71.1 and 73.3%, respectively (Table-5). Results are consistent with reports by other investigators who indicate that ciprofloxacin and the fluoroquinolones, in general, are the most active antituberculosis drugs [34-36]. In contrast, other studies reported that amikacin was a significantly more effective drug when tested against *M. avium* [37].

**Table 4:** The susceptibility of MBTC isolates to some of the first-line antituberculous drug.

Name of the first-line antituberculous drug	Number of tested isolates	Sensitive isolates	

		n	%
Pyrazinamide	135	29	21.5
Isoniazid		55	40.7
Rifampicin		80	59.3
Ethambutol		92	68.2

**Table 5:** The susceptibility of MBTC isolates to some of the second-line antituberculous drug.

Name of the second-line antituberculous drugs	Number of tested isolates	Sensitive isolates	

		n	%
Kanamycin	135	24	17.7
Amikacin		26	19.3
Ciprofloxacin		96	71.1
Clarithromycin		99	73.3

## Conclusion

Multidrug-resistant *Mycobacterium tuberculosis* complex (MDR-TB) was isolated from raw milk and lymph nodes of field tuberculin positive cows and from sputum of veterinarians and workers in contact with these animals at existing farms and abattoirs in three Delta Egypt governorates. Precise and rapid diagnosis of *M. tuberculosis* complex was achieved through application of advanced RT-PCR methods that proved useful for the detection of infected milk, lymph node, and sputum samples and confirmed strains of *M. tuberculosis* complex isolated by conventional methods. RT-PCR can be used for diagnosis of TB either directly in samples or in early cultures, in a single day instead of a 1-2 months period required for identification by culturing. After identification of TB, the use of a combination of first-line antituberculosis drugs, such as EMB, with second-line drugs, such as ciprofloxacin and clarithromycin, might greatly reduce the incidence of *MTBC* isolates in bovines and bovine products.

## Authors’ Contributions

HAA planned the research and experimental design, collected the samples, and performed the laboratory work and data analysis. HMS and KFM supervised the work, checked the data analysis, and revised the manuscript. SHAH helped in the drug susceptibility assay. AND helped in the isolation, identification, and molecular diagnosis through real-time polymerase chain reaction and revised the manuscript. ASH helped in the drug susceptibility assay and wrote the manuscript. All authors contributed to the drafting and revision of the manuscript. All authors read and approved the final manuscript.
